# Time lagged investigation of entrepreneurship school innovation climate and students motivational outcomes: Moderating role of students’ attitude toward technology

**DOI:** 10.3389/fpsyg.2022.979562

**Published:** 2022-08-08

**Authors:** Xuemei Yuan, Rudsada Kaewsaeng-on, Shuai Jin, Marhana Mohamed Anuar, Junaid M. Shaikh, Saqib Mehmood

**Affiliations:** ^1^Financial Department, Jiangsu University, Zhenjiang, China; ^2^Faculty of Humanities and Social Sciences, Prince of Songkla University, Pattani, Thailand; ^3^School of Management, Jiangsu University, Zhenjiang, China; ^4^Faculty of Business, Economics and Social Development, Universiti Malaysia Terengganu, Kuala Terengganu, Malaysia; ^5^Accounting Department, School of Business, Universiti Teknologi Brunei, Jerudong, Brunei; ^6^Faculty of Management Science, International Islamic University, Islamabad, Pakistan

**Keywords:** entrepreneurship, school innovation climate, students’ attitude toward technology, students’ motivational outcome, reinforcement theory of motivation, innovation

## Abstract

Based on the reinforcement theory of motivation, the purpose of this research was to measure the effect of school innovation climate on students’ motivational outcomes, including behavioral engagement, academic self-efficacy, interest, and utility value. Furthermore, the conditional influence of students’ attitude toward technology on the link between school innovation climate and students’ motivating outcomes has been investigated and reported. Data were gathered from the 305 entrepreneurship program students of five different universities located in Wuhan, China. In the SamrtPLS 3.3.3 program, the analysis was carried out using SEM. Results revealed that the school innovation climate has a favorable impact on improving the motivating outcomes of students. Additionally, results also provided support for moderation hypotheses that “students’ attitude toward technology” moderated the relationship between “school innovation climate” and academic self-efficacy. On the contrary, “students’ attitudes about technology,” did not appear to be a significant moderator in terms of enhancing the influence of the “school innovation atmosphere” on the students’ behavioral engagement, interest, and utility value. This study provides key policy and theoretical and practical implications as well as future research avenues for entrepreneurial school managers and education scholars.

## Introduction

Because of today’s highly dynamic contemporary business climate, firms must innovate to distinguish their product and service offerings from those of rivals while also delivering value to their clients. Leaders, entrepreneurs and managers may stimulate innovation by creating internal work environments that encourage and reward it (Mumford, 2000). Innovation in the education sector (especially higher education) is crucial, and teachers have the heavy responsibility of delivering the course content and additional knowledge of the peripheral areas as well (Fischer et al., 2020) and making the students creative and innovative, and competent enough to face the challenges (Crawford et al., 2020). Chan Lin et al. (2006) found that entrepreneurship school administrators employ a variety of initiatives and management tactics to encourage an innovative climate. Previous researchers have termed these types of environments as “innovation climates” or “climates for innovation” (Mathisen et al., 2006; Chen and Hou, 2016). The present research argues that “entrepreneurship school innovation climate” is an essential component that determines the motivational outcomes of students.

Furthermore, in an innovative climate, individuals become more competitive, as well as more creative and imaginative, allowing them to cope with engagement, self-efficacy, interest, and utility value in their academic life in a more effective manner (Antoni, 2005). There is paucity of research studies in education management literature linking school innovation climate with various outcomes of students’ motivations, notably those connected to creativity and innovation. Contrarily, the present research is incremental as it tries to fill up this knowledge gap by arguing that school innovation climate in entrepreneurship schools is an essential factor in students’ motivating consequences, such as their level of behavioral engagement, academic self-efficacy, interest, and utility value.

Students’ behavioral engagement is referred to the degree to which a student demonstrates classroom behaviors such as attention, completing assignments, following instructions, and engaging (Cooper, 2014). In addition to that Lane and Harris (2015), conceived the behavioral engagement as timeliness, intellectual engagement in the classroom, communication with the teachers, and involvement in class. Thus, students attending regularly scheduled sessions, raising multiple inquiries, and contacting teachers about a variety of educational and non-educational activities are deemed behaviorally engaged in their studies (Sawatsuk et al., 2018). Because of the favorable impact of behavioral engagement on student success, a significant quantity of research has been conducted to discover the qualities of schools and classrooms that are related to behavioral engagement (Farooq et al., 2010; Wang and Holcombe, 2010; Yazzie-Mintz and McCormick, 2012; Chiu and Shih, 2020; Ain and Waheed, 2021).

The “students’ academic self-efficacy” refers to the students’ belief in their own capacity to understand and perform the tasks assigned to them by their instructors in the class, irrespective of how challenging the activities are (Gebauer et al., 2020). Individuals’ exceptional performance is facilitated by their high levels of self-efficacy beliefs, which increase their level of dedication, effort, and tenacity (Pintrich, 2003). Earlier scholars explored that there were a variety of factors that influenced students’ academic self-efficacy while they were learning (Waheed, 2010, 2011; Solanki and Xu, 2018; Jam, 2019).

Different activities associated with the use of contemporary technology have been deemed major indicators of students’ interest in academic and non-academic activities in the past, and this has continued to be true (Makransky et al., 2020), they further explained that students’ academic selection objectives (i.e., their job ambitions) are affected by their interests. Lastly, one of the most significant motivating outcomes of students is the utility value, which demonstrates the workable application of the topics learned by the students in the classroom (Shechter et al., 2011; Thipayasothorn et al., 2016; Solanki and Xu, 2018; Waheed and Leisyte, 2020). The present research examines the impact of “school innovation climate” while creating interest in student engagement and considering the utility value.

Furthermore, the present research has taken into account “students’ attitude toward technology” in terms of establishing and boosting motivating outcomes in students. Attitudes have already been studied in relation to student performance, in several previous studies (Waheed and Jam, 2010; Jam et al., 2013, 2014, 2018; Ahmed et al., 2020; Janet et al., 2020). The present research examined the contingent effect of “students’ attitude toward technology” on the relationship between “school innovation climate” and their motivating outcomes by employing the reinforcement theory of motivation. This theory emphasizes the state of mind of each individual, including his or her emotions and feelings (Gordan, 2014). Theoretically, the “school innovation climate” and “students’ attitude toward technology” serve as positive signals to the development of specific motivating effects in students.

Previous research reported failure ratio of Chinese entrepreneurship attempts by young graduates was 90% in 2017 (Wang et al., 2017). Therefore, the alarming situation has become a hot and urgent topic to study school innovation climate and motivational factors of young entrepreneurs in China (Peng et al., 2021). Here is the gap where research thesis of current study has been built. Current Era necessitate for universities and business schools to adopt the innovation and entrepreneurship projects to prepare current graduates and future entrepreneurs to meet the emerging challenges in post COVID-19 times (Zhao and Fang, 2022). Specially in Chinese government has pushed an innovation oriented progressive strategy which facilitates small businesses, universities and research centers to invest in developing innovation, creativity in their environment. Recently, based on this strategic shift many business and entrepreneurship schools have started focusing on innovation (Peng et al., 2021). This situation makes contextual importance and thesis of investigation for school innovation climate and its role in fostering innovation and motivational outcomes of Chinese students. Newman et al. (2020) devised a direction for the prospective investigation that identifies opportunities for researchers to go both theoretically and empirically to enhance literature on innovation climate in different settings. Thus responding to this call for investigation a unique cultural context of Chinese higher education institutions in Wuhan, China, will help to provide empirical evidence for future growth of research in this area. The present research, which is grounded in the reinforcement theory of motivation, seeks to provide answers to the following crucial research questions:

• Are entrepreneurship students’ motivational outcomes favorably influenced by the school’s innovative climate?
• Is there a moderating effect of students’ attitude toward technology on the relationship between their school’s innovation climate and students’ motivating outcomes?

## Literature review

### Theoretical foundation “reinforcement theory of motivation”

Reinforcement theory explains in detail how individuals learn new behaviors and develop their own sense of self-expression. Fundamentally speaking, teachers’ primary responsibility is to raise students’ understanding and teach them how to earn positive reinforcement. Reinforcement, “is a term in operant conditioning and behavior analysis for the process of increasing the rate or probability of a behavior in the form of response by delivery either immediately or shortly after performing the behavior.” The reinforcement theory of motivation emphasizes that individual’s emotional and psychological state of mind, which includes his or her emotions and feelings. Reinforcement theory focuses on the changes that take place in each individual as a result of certain acts or behaviors. So, based on Skinner: “the external environment of the organization must be designed effectively and positively to motivate the employee.” This theory is a strong motivator for influencing people’s actions and behaviors (Gordan, 2014). Based on the reinforcement theory of motivation, the school innovation climate is the motivational source for students, which directs their behavior. This theory works as a strong tool to design the actions and behaviors of students and students develop behavioral engagement, academic self-efficacy, interest, and utility value. Thus linking the usage of theory to the study problem that entrepreneurship failure rates in China are far higher than ratios of developed countries (Zhao and Fang, 2022). In this scenario it becomes an advance to investigate state of motivational outcomes and its linkages with students attitude toward technology in school innovation climate. Recent studies conducted in China related to innovation and entrepreneurship programs have recommended to bridge this research gap (Yang et al., 2022). Hence current study attempts to add value by extending the current literature in the field of entrepreneurship education.

### Entrepreneurship school innovation climate and students’ motivational outcomes

The degree to which a person feels motivated throughout a learning experience has been identified as a significant determinant of performance (Quinn et al., 2021). In the last few years, scholars have started to look into how innovation climates affect people’s work inclinations and behaviors (Bamberger, 2018). An innovation climate has been defined as “shared perceptions at the team or organizational level regarding the extent to which team or organizational processes encourage and enable innovation” (Begley et al., 2006). Newman et al. (2020) conducted a study to find out the benefits and disadvantages of innovation climate and its outcomes, whereas some researchers also found a negative relationship between innovation climate and an outcome like stress (Dackert, 2010). Some other researchers identified a substantial association between innovation climate and individuals’ motivation to perform, such as work attitude, job satisfaction, engagement, and commitment (Lee and Idris, 2017). Additionally, scholars observed a strong positive significant association between innovation climate and individual behaviors such as creative behavior (Jaiswal and Dhar, 2015), and innovative behavior (Antoni, 2005). The present research looked for the influence of school innovation climate on the motivational outcomes of students. Innovation and Entrepreneurship drive pushed by Chinese government in educational institutions and business schools with special focus on entrepreneurship schools has been an emerging move which grabbed attention of many recent researches in post COVID-19 time (Peng et al., 2021; Zhao and Fang, 2022). According to “Fortune magazine” the failure rate of first entrepreneurial attempts in China was recorded as high as 90% in 2017. This scenario demands investigations into school innovation climate and motivational factors of entrepreneurship students in Chinese universities. Thus, providing an ideal contextual case for building theoretical case of current framework under reinforcement theory.

The motivational theory of Maslow (2000) described motivation as energy that originates inside a person and motivates him or her to undertake action. Alternatively, it may be viewed as an individual’s inner state that motivates him or her to engage in specific activity and attain specific objectives (Ostrow and Heffernan, 2018). There are four distinct types of motivational outcome in students considered by this study, which includes their behavioral engagement, academic self-efficacy, interest, and utility value. The behavioral engagement area involves thinking about students’ behavior in the classroom, involvement in school-related events, and motivation in the educational assignment they have been assigned to do (Khan et al., 2012, Khan et al., 2019; Yazzie-Mintz and McCormick, 2012; Shernoff, 2013; Maghnaoui, 2020; Kahil, 2021). Another key aspect impacting academic achievement is one’s sense of self-efficacy in one’s own abilities. In academic self-efficacy, students’ thoughts and mindsets about their capacities to attain educational excellence are discussed. This includes belief in their capacity to complete academic assignments and confidence in their ability to study the contents well, among other things (Schunk and Ertmer, 2000). The term “interest” refers to an individual’s ability to be compelled by anything just on the basis of their own internal feelings. According to Deci and Ryan (1985), interest is “an important directive role in intrinsically motivated behavior in that people naturally approach activities that interest them.” Students who are just beginning to acquire an interest in a topic might benefit from the utility value by encouraging them to return to the material on a regular basis and thereby deepen their understanding (Renninger, 2000; Shahbaz et al., 2016; Sawatsuk et al., 2018; Sulashvili et al., 2020).

Based on the work of Kang et al. (2016), it is discovered that an innovation climate stimulates the innovative behavior of individuals by increasing their enthusiasm for exploring the new dimensions. This study establishes that the school innovation climate stimulates the innovative and creative behavior of students which motivates them toward behavioral engagement, academic self-efficacy, interest, and utility value of innovation. It is supported by the reinforcement theory of motivation that individuals pick up new behaviors due to some motivation. Further, this theory describes that motivation emphasizes the individual’s emotional and psychological state of mind, which includes his or her emotions and feelings (Akpan et al., 2022). Based on this phenomenon, in the present study, the entrepreneurship school innovation climate is the source of motivation for students to develop their behaviors as successful entrepreneurs. In this research, we explored the possibility that when the climate of the school is enriched with an innovation culture, the students are more motivated to design their behavior. As a consequence, students get more involved in their studies, improve their sense of self-efficacy, and develop a passion for the material they are studying. As a result, it is hypothesized that;

**H1:** *There is a positive relationship between the entrepreneurship school’s innovation climate and the students’ motivational outcome, i.e., (a) behavioral engagement, (b) academic self-efficacy, (c) interest, and (d) utility value.*

### Students’ attitude toward technology as a moderator

“Attitude is about a person’s continued evaluations, feelings, liking, or disliking of a particular product, person, or entity” (Ajzen, 1980). The word “attitude” refers to a broad assessment of a very particular behavior characterized by action, aim, context, and period (Vishnumolakala et al., 2017). Thus, it is stated in this research that entrepreneurial students with an attitude toward technology who are exposed to an innovative environment see a boost in their motivation. It is also critical for university administration to be cognizant of students’ attitudes (both current and prospective) since this influences their tastes, preferences, and behavioral intentions with regard to engagement, self-efficacy interest, and utility value (Simiyu et al., 2020). The assumption behind using “students’ attitude toward technology” as a moderator in this research is that individuals have an attitude toward things, goods, people, or entities. A current study on young entrepreneurs from China has also pointed out toward this research gap of potential moderators between school innovation environment and students motivational outcomes (Yang et al., 2022). Thus providing an ample justification to propose and test the role of students attitude toward technology as a potential moderator in this research. Another recent study on the subject of innovation and entrepreneurship has also pointed toward the research gap to investigate students and young entrepreneurs attitude toward technology in future studies in Chinese context (Zhao and Fang, 2022).

The literature indicates that “students’ attitude toward technology” perform an essential part in the advancement of their motivational outcomes and that keeping in view the role of “students’ attitude toward technology in boosting the effects of “school innovation climate” on students’ motivational outcomes has been derived from this literature” (Simiyu et al., 2020). Furthermore, research has reported that “students’ attitude toward technology” have an influence on their emotions, feelings, actions, and behaviors as well (Mazhar et al., 2012; Waheed and Kaur, 2016; Mercader and Gairín, 2020). Additionally, positive attitudes and beliefs help students improve their capacities to attain academic achievement, as well as their confidence in their capacity to complete academic assignments and study the topics successfully (Hayat et al., 2020). Therefore in this respect, the reinforcement theory of motivation, which holds that individual behaviors are regulated by their environment, might aid in understanding the phenomena of attitude as a moderator (Gordan, 2014; Akpan et al., 2022). Researchers have previously debated that when “students’ attitude toward technology” coincides with the innovation atmosphere, it aids more effectively in enhancing motivation and shaping behavior. As a result, it is hypothesized that;

**H2:** *Students’ attitude toward technology moderate association of school’s innovation climate with students’ motivational outcome, i.e., (a) behavioral engagement, (b) academic self-efficacy, (c) interest, and (d) utility value. In case of higher levels of attitude toward technology the influence of school innovation climate will be enhanced.*

### Theoretical framework of the study

**FIGURE 1:**
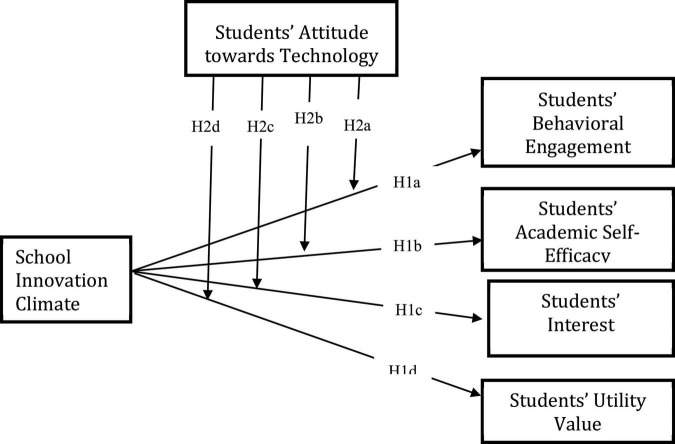
Theoretical framework of the study.

Figure 1 depicts the theoretical framework for the research, which was developed in part from a review of the literature and the reinforcement theory of motivation.

## Research methodology

The population of this study was the students of five higher education universities in Wuhan, China. The context of current research Wuhan, China is the capital mega city of Hubei Province in China with average population of 12 million people and GDP of 224 billion as per 2018 statistics (Yang et al., 2020).

All these higher education universities were running entrepreneurship schools to qualify as a sample for this study. A time-lagged approach with a two-wave survey was used to collect the data through a convenience sampling technique. Respondents were requested to respond to the predictor variable and moderator in the first wave (T1). After 3 weeks in the second wave (T2), responses were recorded for the outcome variables. Convenience sampling was the appropriate technique for this study, as it is a sort of sampling in which the first accessible observational inquiry is utilized for the study without the need for any further data sources to be obtained. Also, it helps to obtain the data relatively in a faster and inexpensive way and participation is open to all participants. Although convenience sampling has been criticized by many researchers (Etikan et al., 2016). The investigation through convenience sampling has been supported along with specific criteria of inclusion (Jager et al., 2017; Kempen and Tobias-Mamina, 2022). The current research has a specific purpose to investigate motivational outcomes of entrepreneurship students in business schools which will be considered a special inclusion criterion. Thus, convenience sampling method can be appropriately used for this research. After obtaining appropriate permissions from the ethical committees of the researchers’ respective institutions, and in accordance with the protocols outlined by Dalle et al. (2021), researchers addressed the administrative departments of their respective universities for further consideration. The study’s goal was explained in detail to administrative departments. Followed by receiving official authorization, students were approached and asked to take part in the survey voluntarily. Students who volunteered to fill out the questionnaire were given that survey to fill it out. It was ensured that students were good in understanding and using English, those who were not expert were excluded from survey by the researchers. The students were assigned a unique ID to identify the questionnaires for compiling after completion of the second phase. The medium English was used to design the questionnaires, as English is well understood in these universities.

In the first phase (T1) the students filled out the questionnaire to rate the predictor variable (entrepreneurship school innovation climate) and moderating variable (students’ attitude toward technology). A total of 450 questionnaires were distributed in the first phase at T1, out of which 355 were received back. The 350 questionnaires were sent back to the same students in the second phase at T2 after 3 weeks from the completion of the first phase, to rate the outcome variables (behavioral engagement, academic self-efficacy, interest, and utility value). Authors received 325 filled questionnaires, some partially filled and unengaged responses were discarded and study left with 305 paired complete paired useable responses with active response rate of 68%.

### Measures of the study

This study devised a research survey to investigate the relationship between “entrepreneurship school innovation climate” and students’ motivational outcomes. The predictor variable ‘school innovation climate’ was measured by adapting a scale having four-item developed by Fraser and Rentoul (1982). Participants were asked to rate at “a 5-point Likert scale ranging from 1 = strongly disagree to 5 = strongly agree.” Authors employed four constructs to assess students’ motivating outcomes: students’ behavioral engagement, students’ academic self-efficacy, students’ interest, and students’ utility value. A scale developed by Solanki and Xu (2018) was employed to assess these constructs. Concurrently, students’ behavioral engagement was assessed by using the four items, “During this course, about how often have you done the following: (a) attended lectures, (b) listened attentively to lectures, (c) asked the professor or TA for help in this class, and (d) asked questions and contributed to the discussion in lecture?.” To measure the responses “a five-point Likert scale was used with 1 = never to 5 = always.” At the same time, academic self-efficacy was measured with three items, i.e., “(a) I am certain I can master the skills taught in this class,” (b) “I’m certain I can figure out how to do the most difficult course material, and (c) I can do almost all the work in the class if I don’t give up.” Moreover, students’ interest was measured with three items, i.e., (a) “I find many topics in this course to be interesting, (b) Solving problems in this class is interesting for me, and (c) I find this class intellectually stimulating.” The students’ utility value for innovation was assessed with two items, i.e., “(a) Having a solid background in the material taught in this course is worthless, and (b) After I graduate, an understanding of the material in this course will be useless to me.” The respondents rated these items of all constructs on “a five-point Likert scale ranging from 1 = not at all true to 5 = very true.” Finally, the attitude toward educational technology was measured with three items adapted from Öztürk (2006) and was later used by Atabek (2020). The sample items include “reflection of using technology in education on instructional processes,” “improving oneself in using technology in education,” and “using technology in education and classroom management.” The responses were measured at “a 5-point Likert-type scale (1 = Strongly Disagree, 5 = Strongly Agree).”

### Demographic characteristics of the respondents

Table 1 show the demographic characteristics of those students who participated and answered the survey questions.

**TABLE 1:** Respondents’ demographic characteristics.

Variables		Students
Gender	Female	43.4%
	Male	56.6%
Age	18–25 years	53.4%
	26–30 years	25.6%
	31–35 years	13.7%
	36 and above	07.3%
Qualification/Degree level	Undergraduate level	51.8%
	MBA/MS/Graduate level	36.5%
	PhD/Post-graduate	11.7%
	Post Doc	–

## Data analysis and results

It was necessary to use SmartPLS 3.3.3 software in order to perform an initial assessment and evaluate the psychometric features of the construct. The findings revealed that “entrepreneurship school innovation climate” had a favorable influence on students’ motivating outcomes. As a result, the “school innovation climate” was taken into consideration throughout the study.

### Measurement model assessment

To determine the integrity of a measurement scale, the validity test was used. In order to determine the validity of the data, Confirmatory Factor Analysis (CFA) was used, which is designed to validate the most dominating components in a set of variables (factor loading). When a standardized factor loading (SFL) of more than 0.70 is found in an indicator, it is considered to have strong validity (Hair et al., 2019). Table 2 depicts the results of outer loading, which is meeting the threshold point.

**TABLE 2:** Outer loadings.

	*SIC*	SAT	SAS	SBE	SII	SVI
SAS1			0.865			
SAS2			0.950			
SAS3			0.814			
SAT1		0.880				
SAT2		0.885				
SAT3		0.904				
SBE1				0.896		
SBE2				0.949		
SBE3				0.898		
SBE4				0.807		
SIC1	0.835					
SIC2	0.918					
SIC3	0.812					
SIC4	0.900					
SII1					0.844	
SII2					0.824	
SII3					0.944	
SVI1						0.902
SVI2						0.933

The validity and reliability of the constructs were tested using “convergent validity,” which includes “Cronbach’s Alpha (CA), rho_A, Composite Reliability (CR), and Average Variance Extracted (AVE)” (Henseler et al., 2015). “Cronbach’s alpha and rho_A” are recommended to be more than 0.7. The “Composite Reliability (CR)” of a variable is determined by a group of indicators that indicates whether or not the variable has strong “Composite Reliability (CR),” defined as higher than 0.7. According to the proposed method, the determined value of “Average Variance Extracted (AVE)” should be higher than 0.50. Table 3 depicts that all the figures meet the threshold point, As a result, “convergent validity” has been established (Hair et al., 2017, 2019).

**TABLE 3:** Construct reliability and validity.

	Cronbach’s alpha	rho_A	CR	AVE
School Innovation Climate	0.890	0.899	0.924	0.752
Students Attitude toward Technology	0.871	0.904	0.919	0.792
Students’ Academic Self-Efficacy	0.879	0.907	0.909	0.771
Students’ Behavioral Engagement	0.921	0.923	0.938	0.790
(Hair et al., 2019) Students’ Interest	0.867	0.887	0.905	0.761
Students’ Utility Value	0.814	0.833	0.914	0.842

CR, composite reliability; AVE, average variance extracted.

As part of SEM “discriminant validity” ensures that a measure of construct is both empirically exclusionary and capable of describing observed phenomena that other measures in the model seem unable to explain (Hair et al., 2010). To put it another way, “discriminant validity” requires that “a test does not correlate too highly with measures from which it is supposed to differ” (Campbell and Fiske, 1959). The discriminant validity of the questionnaire was established by using the (Fornell and Larcker, 1981) approach, which was developed by the authors. This outcome is achieved by having the square root of the AVE greater than the sum of all correlations within the same row and column of the specified construct, as seen in Table 4 given below.

**TABLE 4:** Fornell and Larcker.

	*SIC*	SAT	SAS	SBE	SII	SVI
School Innovation Climate	0.867					
Students’ Attitude toward Technology	0.664	0.890				
Students’ Academic Self-Efficacy	0.163	0.187	0.878			
Students’ Behavioral Engagement	0.103	0.200	0.205	0.889		
Students’ Interest	0.297	0.078	0.130	0.018	0.872	
Students’ Utility Value	0.573	0.241	–0.174	0.038	0.062	0.918

”The Fornell and Larcker (1981) criteria,” which is the most commonly used “discriminant validity criterion,” is ineffectual in specific circumstances (Henseler et al., 2009; Rönkkö and Evermann, 2013), denoting that the quite commonly used “discriminant validity yardstick” may have a shortcoming (Rönkkö and Evermann, 2013). To address this critical issue, Henseler et al. (2015) have devised a new approach for establishing “discriminant validity” that they believe is superior to the existing methods. “The Heterotrait-Monotrait Correlations Ratio (HTMT)” is a novel method for determining “discriminant validity.” In order to ensure that all research constructs are unique, the HTMT ratio was set below 0.90. Table 5 shows that all results are below the HTMT criterion of 0.85.

**TABLE 5:** Heterotrait-Monotrait ratio.

	*SIC*	SAT	SAS	SBE	SII	SVI
School Innovation Climate						
Students’ Attitude Toward Technology	0.738					
Students’ Academic Self-Efficacy	0.164	0.169				
Students’ Behavioral Engagement	0.134	0.196	0.220			
Students’ Interest	0.281	0.137	0.182	0.095		
Students’ Utility Value	0.662	0.271	0.178	0.063	0.060	

”The goodness of fit (GoF)” has been created as an indicator of the overall model fit for PLS-SEM. Nevertheless, because the “GoF” measure cannot consistently discriminate valid from invalid models and because its usefulness is confined to certain model settings, researchers should avoid using it as a GoF metric in their study. When calculating approximation fit indexes such as “SRMR and NFI,” the results of a PLS-SEM model estimate are taken into account, as well as the values of these parameters that meet a particular threshold “(e.g., SRMR 0.08 and NFI > 0.90).” In accordance with Table 6, the GoF of this model has been shown.

**TABLE 6:** Goodness of fit.

	Saturated model	Estimated model
SRMR	0.079	0.079
d_ULS	1.514	2.057
d_G	1.175	1.226
Chi-Square	258.522	265.833
NFI	0.619	0.608

**FIGURE 2:**
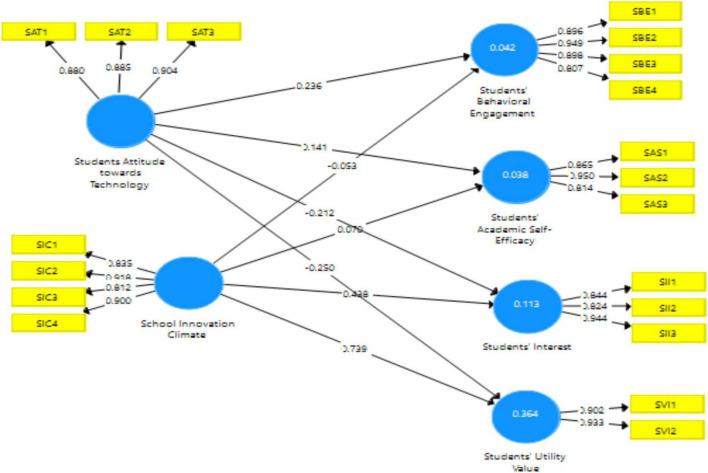
Measurement model.

When it comes to data analysis, the “coefficient of determination” is a complicated concept that is based on statistical modeling. The “coefficient of determination” is a statistical concept that describes the degree to which the link between two variables might affect the variability of one of the variables. This number ranges from 0.0 to 1.0, with 1.0 indicating a perfect fit and consequently a very dependable model for future projections, and 0.0 indicating the model fails to properly describe the data at all. The present study’s findings are being presented 44.1, 45.3, 46.3, and 43.5% variance in students’ behavioral engagement, academic self-efficacy, interest, and utility values. The measurement model is presented in Figure 2 below.

### Structural model assessment

#### Path coefficients

When calculating the structural model, we used the conventional PLS-SEM criteria to ensure that the predicted correlations were not distorted. In order to do this, the conventional bootstrapping approach was utilized in conjunction with 5000 samples in the SmartPLS 3.3.3 program to determine the “path coefficients’ significance level” (Sarstedt et al., 2017; Hair et al., 2019). As explained, path values, the values of R square and adjusted R square are presented in Table 7.

**TABLE 7:** Coefficient of determination (*R*-square).

	*R* Square	*R* Square adjusted
Students’ Academic Self-Efficacy	0.453	0.447
Students’ Behavioral Engagement	0.441	0.437
Students’ Interest	0.463	0.454
Students’ Utility Value	0.435	0.429

#### Hypothesis testing (direct effect)

To evaluate H1a, b, c, d, we first examined the direct influence of independent variables on dependent variables. The findings of the direct connection between variables are shown in the following table. The present research established a statistically significant positive association between the “school innovation climate” and students’ behavioral engagement (Coefficient = 0.937, *p* = 0.05), academic self-efficacy (Coefficient = 0.937, *p* = 0.05), interest (Coefficient = 0.937, *p* = 0.05), and utility value (Coefficient = 0.937, *p* = 0.05). Additionally, Table 8 presents the findings of the direct relationship hypotheses H1a, b, c, and d, indicating that all hypotheses were accepted.

**TABLE 8:** Direct relationships.

Hypothesis		Original sample	Sample mean	*T* statistics	*P*-values	Supported
H1a	*SIC* - > SBE	0.937	0.983	2.025	0.043	Yes
H1b	*SIC* - > SAS	0.952	0.997	1.993	0.046	Yes
H1d	*SIC* - > SII	0.446	0.338	1.654	0.013	Yes
H1d	*SIC* - > SVI	0.993	0.999	2.529	0.011	Yes

*SIC*, School Innovation Climate; SBE, Students’ Behavioral Engagement; SAS, Students’ Academic Self-efficacy; SII, Students Interest; SVI, Students Utility Value.

#### Hypothesis testing (moderation)

**FIGURE 3:**
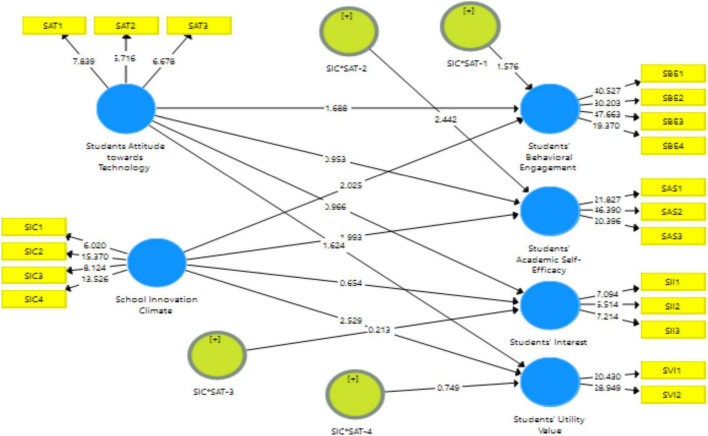
Structural model assessment.

Using the SmartPLS 3.3.3 program, we were able to moderate the connection between independent and dependent variables, as shown in Figure 3. The moderating effect was created by multiplying the moderator by the predictor. Results in Table 9 illustrates that students’ attitude toward technology only moderates the relationship between school innovation climate and students’ academic self-efficacy (Coefficient = 0.895, *p* = 0.05), whereas show no significant results of moderation between school innovation climate and other three dependent variables students’ behavioral engagement (Coefficient = –0.874, *p* = 0.05), interest (Coefficient = –0.079, *p* = 0.05), and utility value (Coefficient = 0.203, *p* = 0.05). Moreover, the results of the moderation hypotheses H2a, b, c, and d are presented in Table 9 reflecting that only one hypothesis was accepted, while the other three were not accepted.

**TABLE 9:** Moderation analysis.

Hypothesis		Original Sample	Sample Mean	*T* Statistics	*P*-values	Supported
H2a	SIC*SAT-1 - > SBE	–0.874	–0.481	*1.576*	*0.115*	No
H2b	SIC*SAT-2 - > SAS	0.895	0.929	*2.442*	*0.015*	Yes
H2d	SIC*SAT-3 - > SII	–0.079	–0.243	*0.213*	*0.831*	No
H2d	SIC*SAT-4 - > SVI	0.203	0.239	*0.749*	*0.454*	No

*SIC*, School Innovation Climate; SBE, Students’ attitude toward technology; SAT, Students’ Behavioral Engagement; SAS, Students’ Academic Self-efficacy; SII, Students Interest; SVI, Students Utility Value.

## Discussion

### Findings

The present research investigates the impact of “entrepreneurship school innovation climate” on students’ motivating outcomes, including “students’ behavioral engagement, students’ academic self-efficacy, students’ interest, and students’ utility value.” Furthermore, the moderating impact of “students’ attitudes toward technology” in boosting the influence of their “school innovation climate” on students’ motivation outcomes was analyzed. The findings indicate that when a school’s atmosphere is more creative and innovative, learners exhibit better behavioral engagement in the educational setting. This also demonstrates that innovative culture may more effectively engage the students to become creative and innovative. These findings are in accordance with the earlier research by Waruwu et al. (2020), which reflects that innovative culture positively impacts behavior and performance.

Additionally, the findings indicate that the “school innovation climate” has a favorable impact on “students’ academic self-efficacy.” This further demonstrates that when the learning atmosphere is creative and innovative, it may help learners acquire the skills they need to grasp the fundamentals of the subject matter. These results also show that a “school innovation climate” may assist students in becoming more creative and innovative, as well as in developing a person’s belief that he/she would be able to attain success at a certain degree in a given academic subject area. These findings are consistent with the study conducted by Novitasari et al. (2020), that capacity to innovate has a favorable and statistically significant impact on performance.

Simultaneously, the findings indicate that the “entrepreneurship school innovation climate” increases students’ interest to study that topic. Students’ desire to be creative and innovative is piqued even more when the educational atmosphere is innovative and supportive, and when the discussions are based on creativity and innovation. These results may be tied to prior research, which demonstrated that an innovation climate encourages employee creativity and they develop an interest in innovation (Jaiswal and Dhar, 2015; Zhao and Fang, 2022).

Furthermore, the findings demonstrate a favorable relationship between “school innovation climate” and “students’ utility value for innovation.” This further demonstrates that the school’s innovative environment can further help students in using their newfound knowledge in their everyday lives and they may place a high value on a future benefit or consequence that it brings about. These findings are consistent with the study conducted by Fidalgo-Blanco et al. (2017), which emphasized the importance of innovation climate in convincing students to believe in it and apply it to their career development.

The study’s findings reveal that, in addition to the direct relationships mentioned above, students’ attitude toward technology have a contingent influence on the link between their “school innovation climate” and their students’ motivating outcomes. The results further revealed that students’ attitude toward technology, only influences one relationship “school innovation climate” and “students’ academic self-efficacy,” whereas does not influence the relationship between “school innovation climate” and “students’ behavioral engagement, students’ interest, and students’ utility value.” These results are quite different from the previous findings, which suggest that students’ attitude toward technology are more helpful in enhancing the students’ performance and motivation outcomes (Cai et al., 2017; Akpan et al., 2022).

Because they are founded on the reinforcement theory of motivation, these results are a useful complement to the current body of knowledge that “school innovation climate” shapes the behaviors of students and they become active in behavioral engagement, academic self-efficacy, interest, and utility value. Furthermore, students’ attitude toward technology, helps the “school innovation climate” to enhance the “students’ academic self-efficacy.” Consequently, the moderating effect of students’ attitude toward technology in the relationship of “school innovation climate” with “students’ behavioral engagement, students’ interest, and students’ utility value” was not significant. It demonstrates that the students’ attitude toward technology does not contribute in boosting the influence of “school innovation climate” on “students’ behavioral engagement, students’ interest, and students’ utility value.” The study compliments the findings of Yang et al. (2022).

### Theoretical implications

The present research, which is based on the reinforcement theory of motivation, has various theoretical implications that are worth discussing. The present research is notable for many reasons. First, it is the first time that the impact of “entrepreneurial school innovation climate” in predicting and improving motivating results among university students in Wuhan, China has been examined. It investigates the relationship between the “school innovation climate” and four distinct students’ motivating outcomes, a relationship that has not been earlier investigated. Further significant addition of this research is the identification of the contingent role played by students’ attitude toward technology in the connection between “school innovation climate” and students’ motivational outcomes. This research makes a significant addition to the current knowledge by demonstrating that students’ attitude toward technology helps “school innovation climate” to enhance “students’ academic self-efficacy.” This research is an attempt to bridge the gap between education management literature, technology and innovation literature and theories. This research attempt has opened several research avenues for future theoretical integration in relevant field.

### Practical implications

The present research makes an important addition to the knowledge of academicians and practitioners in higher education schools, as well as the general public. Focusing on the favorable impact of “entrepreneurship school innovation climate” on students’ motivating accomplishments while learning innovation, educational institutions should create a criterion system for choosing teachers to teach creativity and innovation. Faculty who pass all of the creative and innovative assessments should be given the chance to educate the students in their respective fields. The appropriate training ought to be offered by entrepreneurship schools and business schools and universities as well, with teachers being regularly supplied with the newest resources and possibilities to gain knowledge to further their understanding of creativity and innovation. The students will benefit from their comprehensive understanding of the subject area since they will be better guided. Presently, STEM education is highly valued, and many higher education institutions place a strong emphasis on the adoption of new technologies (Solanki and Xu, 2018; Dalle et al., 2020). Moreover, higher education institutions may also implement professional training initiatives in useful disciplines, such as creativity and innovation, in addition to these vital areas. Furthermore, educational institutions must take into account the environment to enhance the students’ trend toward technology through general awareness. However, the findings of the present investigation revealed that the students’ attitude toward technology along with the “school innovation climate” can enhance the motivation of students toward innovation, especially “students’ academic self-efficacy.” Lastly, the state governing bodies might also promote the potentiality for public institutions to establish a creative and innovative environment via collaboration with private sector organizations. Similar recommendations were also suggested by a recent study on entrepreneurship in Chinese context (Yang et al., 2022). Current research is incremental to shed light on motivational outcomes and attitude of students toward technology as recently Chinese government is spending lot of funds to investigate the factors causing high failure rates of Chinese young entrepreneurs. Thus current research provided key policy insights for the policy makers and scholars in the field of entrepreneurship and innovation and entrepreneurship program. Hence contextual implications of current research are much valuable for entrepreneurs and governing authorities in China.

### Limitations and future research directions

There are certain shortcomings to the present research that should be taken into consideration in prospective research. Firstly, the present research only included university students in Wuhan China. It did not include any other locations. Other territories of China, on the other hand, maybe explored in the future. In this respect, comparison research may be carried out to determine the influence of “entrepreneurship school innovation climate” on the motivating outcomes of students from various states in China, with the results being compared. The present investigation was carried out among institutes of higher education. On the contrary, the impact of “school innovation climate” on a variety of student outcomes may be examined at the secondary and middle school stages in the upcoming research. Lastly, the present study used a time-lagged research design, in which data was obtained from students at two different times spaced by 3 weeks were compared and combined. Ongoing longitudinal studies may be done in the future to avoid the common method bias and increase the generalizability of the findings.

## Data availability statement

The raw data supporting the conclusions of this article will be made available by the authors, without undue reservation.

## Author contributions

All authors listed have made a substantial, direct, and intellectual contribution to the work, and approved it for publication.
